# Expression of endogenous mouse APP modulates β-amyloid deposition in hAPP-transgenic mice

**DOI:** 10.1186/s40478-017-0448-2

**Published:** 2017-06-20

**Authors:** Johannes Steffen, Markus Krohn, Christina Schwitlick, Thomas Brüning, Kristin Paarmann, Claus U. Pietrzik, Henrik Biverstål, Baiba Jansone, Oliver Langer, Jens Pahnke

**Affiliations:** 1 Translational Neurodegeneration Research and Neuropathology Lab, University of Oslo (UiO) / Oslo Universiy Hospital (OUS), Postboks 4950 Nydalen, Oslo, 0424 Norway; 2 University of Lübeck (UzL), LIED, Lübeck, Germany; 3 Leibniz Institute for of Plant Biochemistry (IPB), Halle, Germany; 4 Institute for Pathobiochemistry, University Medical Center of the Johannes-Gutenberg-University, Mainz, Germany; 5 Department of Physical Organic Chemistry, Latvian Institute of Organic Synthesis (OSI), Riga, Latvia; 6 Faculty of Medicine, Department of Pharmacology, University of Latvia (LU), Riga, Latvia; 7 Center for Health & Bioresources, Austrian Institute of Technology GmbH (AIT), Seibersdorf, Austria; 8 Department of Clinical Pharmacology, Medical University of Vienna, Vienna, Austria

**Keywords:** Murine amyloid-beta, Abeta, Transgenic mice, Amyloid precursor protein, Alzheimer’s disease, Amyloidosis

## Abstract

Amyloid-β (Aβ) deposition is one of the hallmarks of the amyloid hypothesis in Alzheimer’s disease (AD). Mouse models using APP-transgene overexpression to generate amyloid plaques have shown to model only certain parts of the disease. The extent to which the data from mice can be transferred to man remains controversial. Several studies have shown convincing treatment results in reducing Aβ and enhancing cognition in mice but failed totally in human. One model-dependent factor has so far been almost completely neglected: *the endogenous expression of mouse APP* and its effects on the transgenic models and the readout for therapeutic approaches.

Here, we report that hAPP-transgenic models of amyloidosis devoid of endogenous mouse APP expression (mAPP-knockout / mAPPko) show increased amounts and higher speed of Aβ deposition than controls with mAPP. The number of senile plaques and the level of aggregated hAβ were elevated in mAPPko mice, while the deposition in cortical blood vessels was delayed, indicating an alteration in the general aggregation propensity of hAβ together with endogenous mAβ. Furthermore, the cellular response to Aβ deposition was modulated: mAPPko mice developed a pronounced and age-dependent astrogliosis, while microglial association to amyloid plaques was diminished. The expression of human and murine aggregation-prone proteins with differing amino acid sequences within the same mouse model might not only alter the extent of deposition but also modulate the route of pathogenesis, and thus, decisively influence the study outcome, especially in translational research.

## Introduction

Aggregation of β-amyloid (Aβ) is a key pathological event in Alzheimer’s disease (AD) [14]. While an increased production and/or aggregation propensity is triggering the accumulation of Aβ in familial forms of AD [32], sporadic cases of AD are characterised by impaired Aβ clearance [25]. In most animal models, the pathological events are initiated by overexpression of mutant human transgenes, namely the amyloid precursor protein (APP) and presenilin 1 or 2 (PS1/2) [29]. Despite the potential interactions, transgenes are generally expressed in addition to endogenous murine proteins. Murine Aβ (mAβ) differs in three amino acids from its human homologue (position 5, 10 and 13) [15]. These sequential changes entail vast alterations in aggregation propensity and toxicity [28, 35, 36]. Mixed fibrils of murine and human Aβ generated in vitro possessed a lower solubility than pure human Aβ fibrils [12]. However, this is completely contradictory to in vivo results, showing that deposits of various transgenic mice are by far more unstable than those of AD patients [15, 16, 21]. Accordingly, overexpression of murine APP (mAPP) in transgenic mice enhanced solubility of aggregates, increased vascular deposition of Aβ but left plaque burden unchanged [15]. The effect of a mAPP knockout is controversial, as a recent study found alterations only in a model with slow development of deposits, while no changes were apparent in a more aggressive amyloidosis model [23]. Considering the decisive role of transgenic models in basic, therapeutic and translational research, the impact of endogenous proteins should receive particular attention. To further elucidate the effect of mAPP in hAPP-transgenic models, we assessed the effect of its co-expression in an established transgenic model of cortical amyloidosis.

## Results

To analyse the pathogenic consequences of mAPP co-expression in transgenic mice, APP/PS1 mice [30] were crossbred with mAPP-deficient mice [39]. Originating animals expressing hAPP with Swedish double mutation (KM670/671NL) and mutant human PS1 (L166P) in absence of mAPP, were used for experiments and referred to as mAPP^0/0^. APP/PS1 transgenic mice with natural expression of murine APP served as control and are referred to as mAPP^+/+^.

### Absence of murine APP promotes deposition of human β-amyloid

**Fig. 1:**
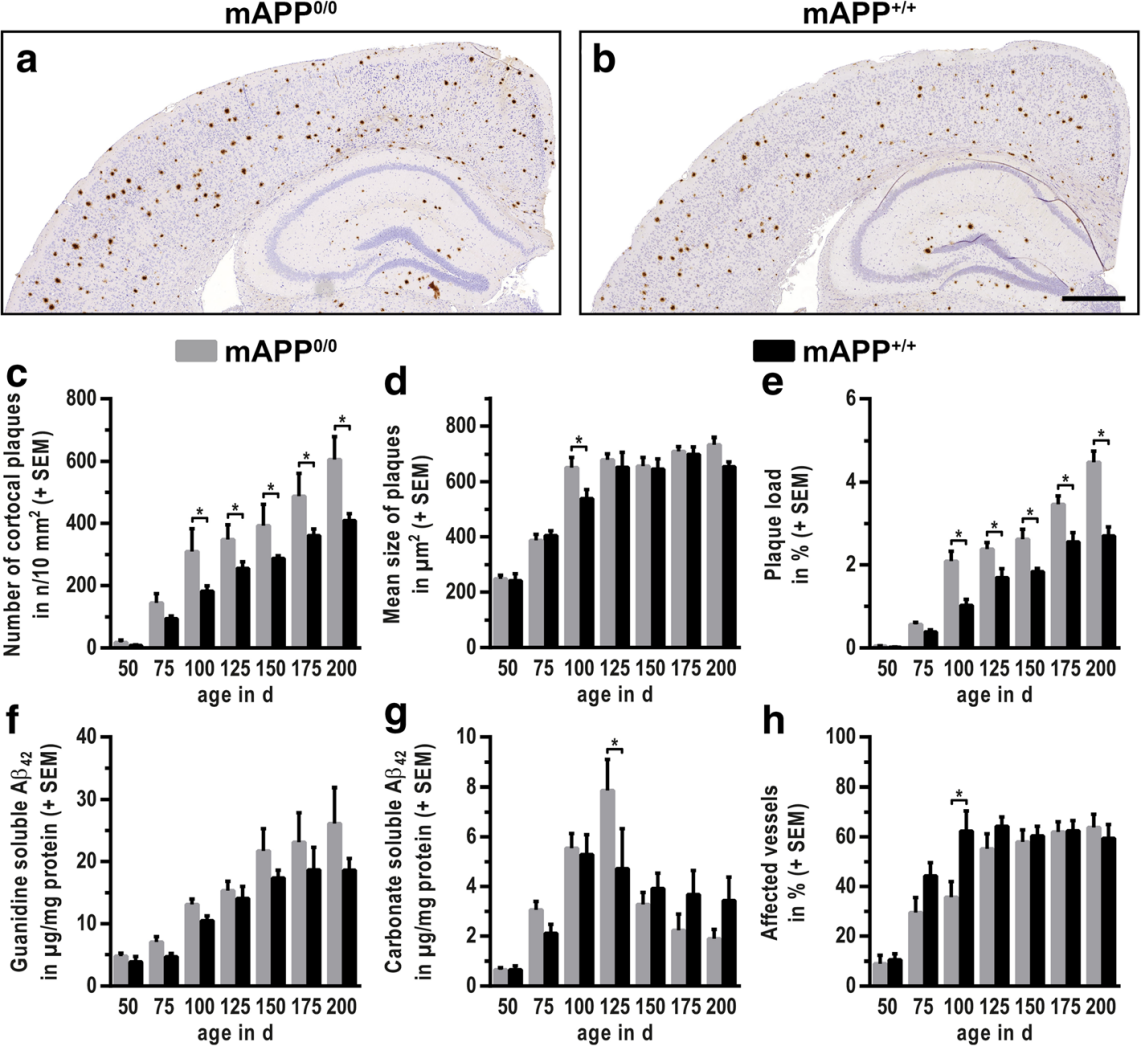
Deposition of β-amyloid is altered upon knockout of murine APP (mAPP^0/0^). Representative micrographs of cortical brain sections from 150 d old **a** mAPP^0/0^ and **b** mAPP^+/+^ mice, immunostained for hAβ and contrasted with haematoxylin illustrate the elevated deposition of human Aβ in murine APP-deficient mice. Semi-automatic analysis confirmed the increase in **c** amount of cortical plaques, **d** mean size of plaques and **e** plaque load (Aβ-positive cortical area). Immunoassays revealed **f** consistently higher levels of deposited (guanidine soluble) Aβ in mAPP^0/0^ mice, while **g** soluble (carbonate soluble) Aβ was only significantly changed at 125 d. In contrast to parenchyma, **h** deposition in leptomeningeal vessels was delayed in murine APP-deficient mice. (Scale bar: 500 μm; * *p* ≤ 0.05; *n* ≥ 7)

**Fig. 2:**
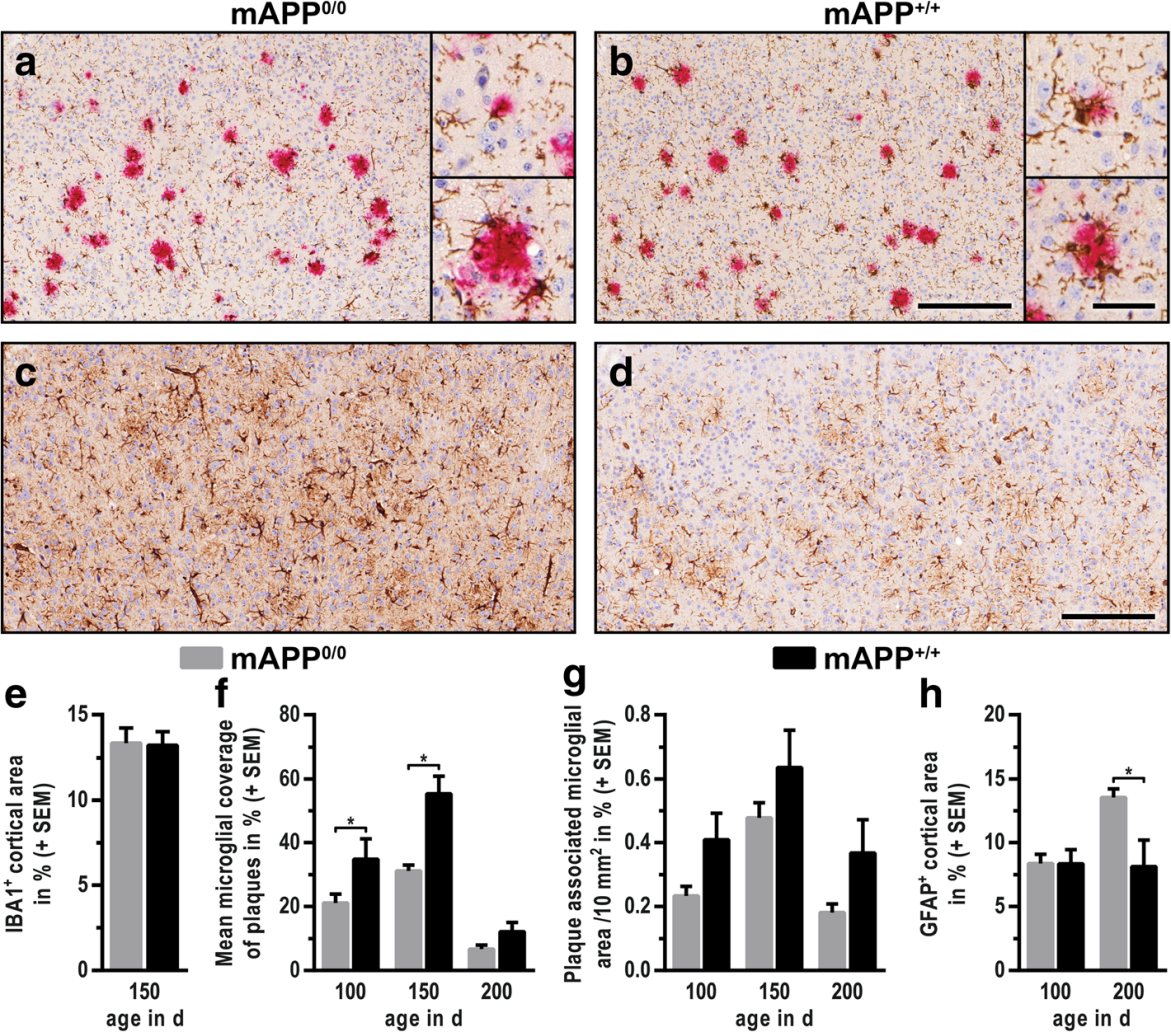
Altered cellular response in murine APP-deficient mice. Cortical brain sections were immunostained for Aβ and IBA1 (**a**, **b**, 150 d) or GFAP (**c**, **d**, 200 d) and contrasted using haematoxylin. Representative micrographs emphasize the impaired microglial response and pronounced astrogliosis in mAPP^0/0^ mice **a**, **c** compared to mAPP^+/+^ animals **b**, **d**. While the total area of microglial cells (IBA1^+^) was stable upon knockout of murine APP **e**, microglial coverage of plaques **f** and the total area of plaque associated microglial cells (G) were reduced. By contrast, a pronounced and age-dependent astrogliosis developed in mAPP^0/0^ mice (C, D, H). (Scale bars: 250 μm in overview, 50 μm in enlargements; * for *p* ≤ 0.05; *n* ≥ 7)

Brain sections of mAPP^0/0^ and mAPP^+/+^ mice were immunostained for human Aβ (hAβ) to screen for qualitative and quantitative differences in cortical amyloidosis. First deposits appeared similar at 50 days of age in both, mAPP^0/0^ and mAPP^+/+^ mice (Fig. 1). However, with increasing age mAPP^0/0^ mice presented with a significantly higher number of cortical deposits compared to mAPP^+/+^ animals (Fig. 1c). While plaque load (Aβ-positive cortical area) was elevated as well (Fig. 1e), the mean size of plaques was just slightly altered (Fig. 1d). Because histological assessment of plaques is only an approximate indication for the amount of deposited Aβ, immunoassays were additionally performed. Two fractions were generated and individually analysed, to distinguish monomers and small oligomers (carbonate-soluble fraction) from fibrillary forms of Aβ (guanidine-soluble fraction). The employed assay recognises both, murine and human Aβ_42_. Levels of aggregated (guanidine-soluble) Aβ_42_ were consistently higher in mAPP^0/0^ mice (Fig. 1f). The amount of carbonate-soluble Aβ_42_ followed a similar pattern with advancing age in both groups (Fig. 1g). To verify that neither altered generation nor degradation provoked the elevated levels of Aβ, expression levels of the most important proteases were determined. Levels of α-secretase ADAM10, β-secretase BACE1 and amyloid degrading IDE were neither age- nor genotype-dependently changed within the analysed age range (Fig. 2).

### Vascular deposition of hAβ is accelerated by co-expression of murine APP

To examine the influence of mAPP expression on the development of cortical amyloid angiopathy, deposition of hAβ in leptomeningeal blood vessels was quantified as previously described [20]. Despite the higher cortical levels of hAβ, deposition in blood vessels was delayed in mAPP^0/0^ mice (Fig. 1h). In general, the proportion of affected vessels strongly rose in both groups until 100–125 days, when a similar and rather stable level was reached.

### Cellular response to hAβ deposits is altered upon knockout of endogenous mAPP

**Fig. 3:**
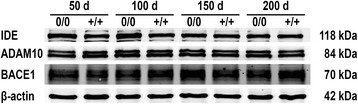
Expression levels of APP- and Aβ-cleaving enzymes remain unchanged. Western blots of the most relevant α- and β-secretases (ADAM10 and BACE1) revealed an age- and genotype independent expression. Levels of Aβ-degrading IDE were likewise unchanged. (β**-**actin was used as loading control)

The deposition of Aβ activates microglia and astrocytes, which gather in the vicinity of plaques and interfere with further accumulation. At first, microglial reaction was analysed at 150 days of age. Cells were typically organised in clusters and presented with enlarged cell bodies and short, sparsely ramified processes (Fig. 3). The total area covered by microglial cells was identical in both groups (Fig. 3e). To explore potential differences in microglia-plaque interaction, their interplay was analysed in hAβ and Iba1 double-stained sections. Microglia were largely found to surround amyloid plaques, whereby their respective coverage depended on age and the particular size of plaques. The mean coverage of plaques by microglial cells was diminished upon knockout of endogenous mAPP (Fig. 3f). To evaluate if the decreased response was solely caused by elevated plaque load, the total area of plaque-associated microglia was determined and found to be lower in mAPP^0/0^ mice as well (Fig. 3g). Sections immunostained for GFAP were analysed to detect astrocytic alterations. While total area of GFAP-positive astrocytes remained unchanged during aging in mAPP^+/+^ mice, mAPP^0/0^ animals developed a pronounced and age-dependent astrogliosis (Fig. 3).

### Neuronal density is unaffected by endogenous mAPP knockout

**Fig. 4:**
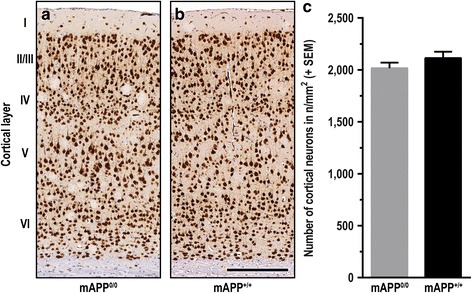
Neuronal density is unchanged in murine APP-deficient mice. Representative brain sections immunostained for NeuN (neuronal nuclei) and contrasted using haematoxylin revealed no significant differences in neuronal density between mAPP^0/0^ **a** and mAPP^+/+^ mice **b** at 150 d. Semi-automatic evaluation of digitised slides confirmed similar density of neurons in both groups **c**. (Scale bar: 250 μm; unpaired t**-**test with Welch’s correction displayed no significant differences; *n* ≥ 10)

**Fig. 5:**
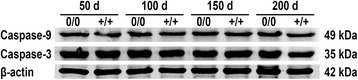
Expression levels of major caspases remained unchanged. Western blot analysis indicated neither age- nor genotype-dependent differences between in expression levels of caspase-3 and -9. (β**-**actin was used as loading control)

Progressive neuronal loss and apoptosis are characteristic but relatively late events in the pathogenesis of AD. To determine the effects of murine APP co-expression on the number of cortical neurons, NeuN-stained sections were analysed. Despite the higher hAβ load in mAPP^0/0^ mice, the neuronal density in layers II to VI was not significantly changed at 150 days of age (Fig. 4). Additionally, we analysed expression levels of two caspases which contribute to neuronal death and show abnormal expression levels in patients with AD, initiator caspase 9 and effector caspase 3. However, protein levels displayed neither age- nor genotype-dependent differences and therefore as well no hint for alterations in apoptosis within the analysed age range (Fig. 5).

## Discussion

Most of the current murine models of AD co-express mutant human transgenes to induce the desired pathology. However, potential interactions between endogenous and transgenic APP proteins have rarely been addressed so far. By combining an murine model of cortical amyloidosis [30] with a murine APP knockout strain [39], we have shown here that mAPP co-expression significantly alters accumulation and aggregation of hAβ. Mice that exclusively express hAPP developed more amyloid deposits and presented with higher levels of aggregated hAβ_42_, while deposition in cortical blood vessels was delayed, levels stabilized and were equivalent in older animals. The inverse changes in soluble and aggregated hAβ_42_ levels further indicated an alteration in general solubility.

In line with our results a previous study showed that additional overexpression of mAPP in APP/PS1 double transgenic mice elevated the solubility of generated deposits and intensified accumulation in cortical blood vessels, but neither accelerated nor increased the parenchymal deposition of hAβ [15]. Another recent study suggested that the impact of mAβ on aggregation depends on the particular model, as deposition was only changed when amyloidosis developed slowly [23].

Murine Aβ is generally thought to contribute to amyloid load, as it accumulates in transgenic models [23], generates mixed fibrils with human Aβ [12] and is part of amyloid plaques in transgenic animals [23, 37]. Although APP gene dose is principally of crucial importance [13], neither physiological expression [23] nor overexpression [15] of mAPP increased plaque load. The contributing role of mAβ in hAPP-transgenic models might, therefore, no longer remain sustainable. Furthermore, aggregating proteins implicated in other neurodegenerative disorders possess similar characteristics. In mice, endogenous tau was shown to interfere with aggregation of transgenic human tau [1]. In humans, mutant variants of hAPP [3] and PrP [2] protect their carriers from aggregation and consequently development of either AD or Kuru and classical Creutzfeldt–Jakob disease.

Although mAPP-deficient mice are viable and fertile, they display a number of relevant abnormalities. They present with a reduced body and brain weight [22] and diminished locomotor activity [7, 34]. Furthermore, these mice develop a pronounced and age-dependent astrogliosis and show impairments in spatial learning and memory [7, 27, 34]. The partial compensation by transgenic hAPP that might be speculated is likely restricted by the utilised Thy1-promotor, which is activated postnatally in neuronal cells [30]. Herein, we used the C57BL/6 J genetic background for experiments, as it was shown to reduce the adverse effects of APP-deficiency [22].

Our findings confirm the previously described age-dependent astrogliosis [7]. Due to the variety of astrocyte functions, the impact of the astrogliosis can hardly be estimated under these conditions. But as astrogliosis typically reduces plaque load in murine AD-models [5, 6], the observed alterations are unlikely the cause for the increased deposition of Aβ. However, astrocytes were further shown to suppress the recruitment and activation of microglia in APP/PS1 transgenic animals [19]. Accordingly, we found a substantial reduction in microglial plaque coverage upon knockout of murine APP that might be triggered by the strong astrogliosis. As microglia actively clear soluble [24] and fibrillary [18] Aβ, their diminished recruitment might further contribute to elevated levels of Aβ.

Due to the important functions of APP in development and maintenance of the brain, mAPP^0/0^ mice additionally suffer from the lack of beneficial APP functions in neuronal development. However, neither neuronal density nor expression levels of caspases were significantly altered in the analysed age range. This corresponds to previous studies, showing that neuronal loss is not evident until 8 months of age in the utilised strain [30].

The majority of therapeutic strategies against AD are directed against Aβ. The accurate reconstruction of Aβ aggregation is therefore of paramount importance. A decreased aggregation propensity, analogical to the herein reported effect of murine APP co-expression, not only restricts accumulation and aggregation but makes Aβ better available for degradation [3] and promotes its elimination. The extent of aggregation interference, peripheral and central degradation and the efflux across the blood-brain barrier by LRP1 [17, 38] and different ABC-transporters [20, 26] might, therefore, been estimated inaccurately. The co-expression of murine APP may, thus, *impede transferability* of results to the human system.

To eliminate the interactions between transgenic hAβ and endogenous mAβ, humanised models can be used as background strains. Although the mutagenesis of mAPP has early been reported [8, 31], it attracted only little interest. While humanisation of mAPP already increased the production of Aβ, it did not provoke deposition of Aβ [8]. However, a more physiological murine model of inherited AD with slow developing amyloidosis was generated by targeted mutations in humanised APP (KM670/671NL) and PS1 (P264L) [9]. This model achieves Aβ deposition without any overexpression. Furthermore, keeping *APP* and *PSEN1* in their chromosomal position with the natural promotor preserves the developmental, cell- and tissue-specific expression pattern of APP and PS1.

## Conclusions

The vast majority of the employed murine models of AD are premised on the overexpression of mutant human transgenes to provoke the desired pathological changes. Combining a murine APP-deficient and a human APP-transgenic strain, we were able to analyse the progression of the cortical amyloidosis independent of the murine variant. The lack of endogenous mAPP resulted in accelerated deposition and, thus, increased number of senile plaques and higher levels of aggregated hAβ. The delayed deposition in cortical blood vessels further substantiates the assumption of an altered aggregation propensity of hAβ in the presence of endogenous mAβ. The depletion of mAPP also modulated the balance of astrocytic and microglial response, as a pronounced and age-dependent astrogliosis develops, which was accompanied by a diminished microglial association to amyloid plaques. In summary, our results indicate that the coexpression of endogenous mAPP with transgenic hAPP has a significant effect on the deposition of hAβ in the analysed transgenic model of AD. Research results and treatment studies to date might have, therefore, been affected by the interference of the endogenous murine APP, depending on the employed model and the specific experiments. Such interspecies effects should also be kept in mind when dealing with models for other diseases caused by aggregation-prone proteins.

## Materials and methods

Chemicals and materials were purchased from Carl Roth GmbH (Germany), unless stated otherwise.

### Animals

Inbred C57BL/6 J mice provided genomic background for all analysed mice and were purchased from the Jackson Laboratory (C57Bl/6 J, #000664). APP-knockout mice [39] were purchased as congenic strain in the C57Bl/6 J genomic background from the Jackson Laboratory (B6.129S7-Apptm1Dbo/J, #004133). Transgenic C57Bl/6 J mice harbouring two mutant human transgenes, amyloid precursor protein (KM670/671NL) and presenilin 1 (L166P) both driven by the murine Thy1.2-promoter [30] (B6-Tg(Thy1-APPswe; Thy1-PS1 L166P)) were used as model for cortical amyloidosis and were kindly provided by the University of Tübingen, Germany. Heterogeneous, APP/PS1 transgenic mice with natural expression of murine APP (APP/PS1^+/0^, mAPP^+/+^) were used as controls throughout all analyses and are referred to as mAPP^+/+^. To induce cortical amyloidosis in mAPP-deficient mice, homozygous mAPPko females were mated with heterozygous male hAPP/PS1 mice. Murine APP-deficient mice with human APP/PS1 transgenes (APP/PS1^+/0^, mAPP^0/0^) were used for all experiments and are referred to as mAPP^0/0^. Animals were genotyped to determine actual genetic status of transgenes and targeted mutations. All mice were group-housed in 12-h day/night cycles at 22 °C with free access to food and water. All experiments were approved by local authorities of the state Saxony-Anhalt. A sum of at least seven animals of both genders was used per group and time point.

### Tissue preparation

For tissue preparation, mice were sacrificed by cervical dislocation and transcardially perfused with PBS. The brain was removed; one hemisphere was stored in buffered 4% paraformaldehyde for paraffin-embedding and immunohistochemistry, while the other hemisphere was snap-frozen in liquid nitrogen and stored at −80 °C for biochemical analysis [11].

### Immunohistochemistry

Tissue was post-fixed in 4% buffered paraformaldehyde solution for 72 h, dehydrated and embedded in paraffin as previously described [10, 33]. 4 μm coronal sections (1.5 mm caudal of bregma) were mounted, deparaffinised and rehydrated before peroxidase-blocked and immunostained using BOND-III Autostainer. Epitope retrieval was carried out as follows: 5 min in 95% (*v*/v) formic acid for 6F3D; 20 min in EDTA buffer (1 mM EDTA, 0.05% (*v*/v) Tween 20, pH 9.0) for IBA1; 10 min enzymatic digestion (Bond Enzyme Pretreatment Kit) for GFAP or 20 min in citric acid buffer (10 mM citric acid, 0.05% (*v*/v) Tween 20, pH 6.0) for NeuN. Antibodies against β-amyloid (clone 6F3D; Dako Deutschland GmbH, Germany; 1:100, 15 min), ionised calcium-binding adapter molecule 1 (IBA1; Wako Chemicals, Germany; 1:1000, 15 min), glial fibrillary acid protein (GFAP; Dako Deutschland GmbH, Germany; 1:500, 15 min), neuronal nuclear antigen (NeuN; Millipore, Germany; 1:500, 15 min) were used and detected with Bond Polymer Refine Detection kit (Leica Biosystems GmbH, Germany). For double-stained slides, Aβ was detected on the same slide as Iba1 using anti-β amyloid clone 6F3D (1:100, 15 min) and the Bond Polymer Refine Red Detection kit (Leica Biosystems GmbH, Germany). Finally, all slides were counterstained (haematoxylin, 5 min) subsequently dried and embedded using Pertex® mounting medium (Leica Biosystems GmbH, Germany). Slides were digitised using Pannoramic MIDI digital slide scanner (3DHistech Ltd., Hungary) at a resolution of 230 nm/pixel and neocortical areas were analysed under blinded conditions, computer-assisted using either AxioVision (hAβ, GFAP, IBA1 and IBA1/hAβ double stains, Zeiss Microsystems GmbH, Germany) or ImageJ (NIH, USA) (NeuN stains) and the ITCN plugin [4].

### Western blot

Frozen hemispheres were gently thawed in 500 μl RNAlater® (Thermo Fisher Scientific Inc., USA) for one hour on ice, and then were homogenised for 30 s with a homogeniser (SpeedMill PLUS, Analytik Jena AG, Germany) after removing RNAlater®. For western blot analyses, homogenates were dissolved in RIPA buffer with proteinase inhibitors (complete-mini; Roche Diagnostics) and homogenised again. After centrifugation, the protein concentration of the supernatant was determined (Pierce™ BCA Protein Assay; SpectraMax Paradigm, Molecular Devices LLC., USA). Samples were mixed with protein sample buffer (0.25 μl/μl sample, 200 mM Tris, 40% (*v*/v) glycerine, 16% (*w*/*v*) SDS, 4% (*v*/v) 2-Mercaptoethanol) and denatured (5 min, 95 °C). 25 μg protein of each sample was loaded on a 12% polyacrylamide gel. After separation, proteins were blotted onto a PVDF membrane in PAGE transfer buffer (192 mM glycine, 25 mM Tris, 20% (*v*/v) methanol). The blot was blocked (blocking buffer, Rockland Immunochemicals; 1 h, room temperature) and subsequently probed using either anti-ADAM10 (Abcam plc., UK; 1:500), BACE1 (Abcam plc., UK; 1:1000), anti-caspase-3 (Cell Signaling Technology Inc., USA; 1:1000), anti-caspase-9 (Cell Signaling Technology Inc., USA; 1:1000) or anti-Insulin-degrading enzyme (Abcam plc., UK; 1:50) and anti-β-actin (Sigma-Aldrich Co. LLC., USA; 1:30,000). Primary antibodies were diluted in blocking buffer and incubated overnight with gentle agitation at 4 °C. IRDye®-labelled anti-mouse and anti-rabbit antibodies (LI-COR Biosciences), diluted in blocking buffer (1:15,000) were used for detection and incubated for 1 h at room temperature with gentle agitation. Blots were visualised using the Odyssey infrared imaging system (LI-COR Biosciences).

### Electrochemiluminescence immunoassay

For Aβ_42_ electrochemiluminescence immunoassays, brain homogenates were mixed with 20 μl/mg carbonate buffer (100 mM Na_2_CO_3_, 50 mM NaCl, protease inhibitors, pH 11.5); and re-homogenated (SpeedMill PLUS). After centrifugation (20,000 g, 20 min, 4 °C), supernatant and pellet were processed separately. The supernatant was mixed with 610 μl/mL guanidine buffer I (8.2 M guanidine hydrochloride, 82 mM Tris, pH 8.0), rigorously vortexed, centrifuged (20,000 g, 20 min, 4 °C) and supernatant was kept as carbonate soluble fraction. The pellet was mixed with 8 μl/μg protein of guanidine buffer II (5 M guanidine hydrochloride, 50 mM Tris, pH 8.0) and incubated for 3 h (1500 rpm at room temperature), subsequently centrifuged (20,000 g, 20 min, 4 °C) and supernatants were referred to as guanidine-soluble fraction. Protein concentration was determined using ScanDrop® spectrophotometer (Analytik Jena AG, Germany). Aβ_42_ concentration in carbonate- and guanidine-soluble fractions was determined using V-PLEX Aβ42 Kits (Meso Scale Diagnostics LLC, USA) and a MESO QuickPlex SQ 120 (Meso Scale Diagnostics LLC, USA).

### Statistics

Results were statistically analysed using GraphPad Prism 6 (GraphPad Software Inc., USA) and two-way ANOVA followed by Holm-Šidák’s multiple comparison test, were applicable, or unpaired t-test with Welch’s correction and considered significant if *p* ≤ 0.05. Data are presented as arithmetic mean with corresponding standard error of the mean (SEM).
